# Inhibition of Mixed Biofilms of *Candida albicans* and Methicillin-Resistant *Staphylococcus aureus* by Positively Charged Silver Nanoparticles and Functionalized Silicone Elastomers

**DOI:** 10.3390/pathogens9100784

**Published:** 2020-09-25

**Authors:** Humberto H. Lara, Jose L. Lopez-Ribot

**Affiliations:** Department of Biology and South Texas Center for Emerging Infectious Diseases, The University of Texas at San Antonio, San Antonio, TX 78249, USA

**Keywords:** *Staphylococcus aureus*, *Candida albicans*, mixed fungal/bacterial biofilms, catheter infections, silver nanoparticles

## Abstract

Both bacterial and fungal organisms display the ability to form biofilms; however, mixed bacterial/fungal biofilms are particularly difficult to control and eradicate. The opportunistic microbial pathogens *Candida albicans* and *Staphylococcus aureus* are among the most frequent causative agents of healthcare-acquired infections, and are often co-isolated forming mixed biofilms, especially from contaminated catheters. These mixed species biofilms display a high level of antibiotic resistance; thus, these infections are challenging to treat resulting in excess morbidity and mortality. In the absence of effective conventional antibiotic treatments, nanotechnology-based approaches represent a promising alternative for the treatment of highly recalcitrant polymicrobial biofilm infections. Our group has previously reported on the activity of pure positively charged silver nanoparticles synthesized by a novel microwave technique against single-species biofilms of *C. albicans* and *S. aureus*. Here, we have expanded our observations to demonstrate that that silver nanoparticles display dose-dependent activity against dual-species *C. albicans*/*S. aureus* biofilms. Moreover, the same nanoparticles were used to functionalize catheter materials, leading to the effective inhibition of the mixed fungal/bacterial biofilms. Overall, our results indicate the potent activity of silver nanoparticles against these cross-kingdom biofilms. More studies are warranted to examine the ability of functionalized catheters in the prevention of catheter-related bloodstream infections.

## 1. Introduction

Healthcare-acquired infections (HAIs) represent a major problem for hospitalized patients in terms of morbidity, healthcare costs, and in-hospital mortality, particularly for patients in intensive care units (ICUs) [1]. Contaminated health care surfaces in hospitals act as a reservoir for the transmission of pathogens. Medical treatments often depend on sterile medical devices of which catheterization is one of the most common bedside procedures undertaken in hospitals [2]. Catheters are medical devices used in the transfer of body fluids and intravenous drug administration. Polymeric materials (e.g., silicone or polyurethane) are widely used to fabricate catheters. Catheter-related bloodstream infections are the cause of one-third of all HAI-related deaths [3]. Two major problems with implanted catheters are clotting and infection [4], as the use of indwelling medical devices (IMDs) in hospitalized patients offers favorable conditions for microbial biofilm formation leading to the increase of medical device-related infections (DRI) [5]. Biofilms account for over 80% of all DRIs in hospitalized patients [6]. Many bacterial and fungal species are capable of colonizing indwelling catheters as biofilms, causing biofilm dispersal-induced bloodstream infections. The opportunistic pathogenic fungus *Candida albicans* is the fourth most prevalent pathogen that cause nosocomial bloodstream infections (BSI) in the United States, with high mortality rates [7]. On the other hand, methicillin-resistant *Staphylococcus aureus* (MRSA) is a gram-positive multidrug-resistant bacterium responsible for a significant number of hospital-acquired (HA-MRSA) and community-acquired (CA-MRSA) infections that have progressively increased worldwide. MRSA strains display multiple virulence factors, including adhesins, toxins, enzymes, immunomodulatory molecules, and a variety of antibiotic resistance genes. These multiple virulence factors coupled with the ability to form biofilms on indwelling medical devices (IMDs) [8] have made MRSA an important public health burden and the most common cause of hospital acquired bacterial infections. It is estimated that at least 27% of all nosocomial *C. albicans* bloodstream infections (BSIs) resulted in polymicrobial blood cultures, and interestingly *S. aureus* represents the third most frequently co-isolated bacteria mixed with *C. albicans* [9]. These mixed biofilm infections can be difficult for physicians to diagnose, and thus to implement a specific medical treatment often requiring several rounds of antibiotics [10]. In particular, mixed bacterial/fungal biofilms formed by these two species represent a complication of considerable clinical relevance [11]. Typically, *C. albicans* forms highly structured fungal biofilms attached to biotic or abiotic surfaces, and *S. aureus* preferentially adheres to the hyphal elements of *C. albicans* within the biofilm, leading to symbiotic relationship. *S. aureus* resistance to antibiotics is enhanced within the polymicrobial biofilm [9] and moreover, drug resistance to vancomycin has been reported [9]. Biofilm-embedded microorganisms in a matrix of extracellular polymeric substances (EPS) provides higher multi-drug resistance, enhancing its ability to evade the host’s immune response [12]. *C. albicans*-secreted β-1,3-glucan was identified as the key constituent providing the bacteria with enhanced drug tolerance [11] by reducing the diffusion of the antibiotics within the biofilm. Other products by the *C. albicans* biofilm like extracellular DNA [13] and farnesol [14] also raise the drug resistance of *S. aureus*. Furthermore, *C. albicans* facilitates the invasion across the mucosal barriers of *S. aureus*, leading to systemic infection in co-colonized patients [9,15]. Overall, the combined effect of mixed fungal/bacterial biofilms results in an infectious synergism [16].

Silver nanoparticles (AgNPs) are broad spectrum biocides inhibiting viruses [17,18], bacteria [19,20], and parasites [21,22]. Our group previously reported on the potent activity of positively charged AgNPs against single-species biofilms formed by *C. albicans*, mostly via cell wall disruption [23], and those formed singly by MRSA, by thinning and permeabilization of the bacterial cell wall, destabilization of the peptidoglycan layer, and subsequent leakage of intracellular content, causing bacterial cell lysis [20].

Expanding upon our previous observations, here we demonstrate the activity of AgNPs against dual-species *C. albicans*-MRSA biofilms. Moreover, we demonstrate that functionalizing the surface of silicone elastomer sheets with AgNPs results in the inhibition of the formation of these cross-kingdom biofilms. 

## 2. Results

### 2.1. Dose-Response Activity of AgNPs against Mixed Biofilms of C. albicans and MRSA

**Figure 1:**
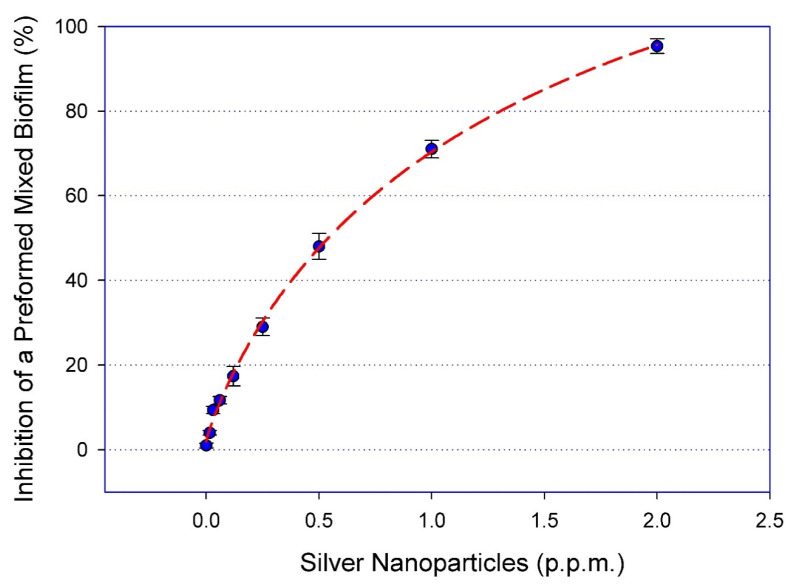
AgNPs display dose response inhibitory activity against mixed *C. albicans*/MRSA biofilms. The efficacy of AgNPs was demonstrated using serial two-fold serial dilutions in concentrations ranging from 2.0 to 0.015 ppm against the preformed mixed fungal/bacterial biofilms in 96-well microtiter plates. The IC_50_ was calculated as AgNPs 0.53 ppm. Curve fitting was performed using the SigmaPlot^®^ plot by four-parameter logistic nonlinear regression equation. Error bars represent standard deviation (SD) of the means.

We have previously reported on the potent in vitro activity of this type of AgNPs against single-species biofilms formed by either *C. auris* [24], *C. albicans* [25], or MRSA [20] alone. Due to the intrinsic recalcitrance of mixed fungal/bacterial biofilms against conventional antibiotic treatment, we were interested in examining the in vitro activity of AgNPs against these cross-kingdom biofilms. Mixed *C. albicans*/MRSA biofilms were grown on the bottom of wells of 96-well microtiter plates, and the preformed mixed biofilms were exposed to a range of concentrations of AgNPs. Our results demonstrated a potent dose-response activity against these fungal/bacterial biofilms, with a calculated IC_50_ value of 0.53 ppm or 530 ng/mL (Figure 1).

### 2.2. Ultrastructural Effects of AgNPs on Mixed Biofilms Using Scanning Electron Microscopy (SEM)

**Figure 2:**
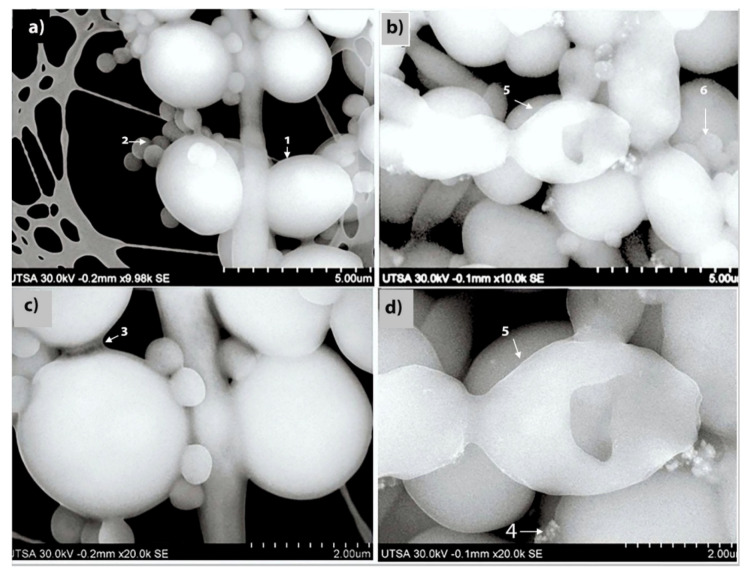
SEM to visualize the effects of AgNPs against dual-species biofilms of *C. albicans* and MRSA. To visualize the ultrastructural effect of AgNPs on the preformed cross-kingdom biofilms, we used advanced SEM techniques. (**a**,**c**) SEM images of preformed mixed biofilms without AgNPs treatment, fungal (1) and MRSA (2) cells are well- defined, with a smooth surface, oval shaped, and showing abundant extracellular polymeric substances of the biofilm (3). (**b**,**d**) demonstrate the changes on the preformed mixed biofilm after AgNPs (4) 24 h treatment (0.53 ppm) presenting alteration, and disruption on the *C. albicans* yeast outer cell membrane (5) and fewer MRSA cells (6). Magnification levels are indicted by the bars at the bottom right of each panel.

An advanced SEM technique was performed to report the ultrastructural effects of AgNPs against mixed biofilms of *C. albicans* and MRSA (Figure 2). The preformed biofilm without AgNPs (Figure 2a,c) show characteristic MRSA cocci cells attached to the fungal elements, both yeast and hyphal cells, with *C. albicans* cells displaying a smooth cell surface. The biofilm exopolymeric substances were also clearly visible (Figure 2a,c). This was in stark contrast to the SEM images obtained after 24 h treatment of the mixed fungal/bacterial biofilms with AgNPs (at a concentration of 0.53 ppm), demonstrating changes in both the shape and surface appearance of the fungal cells, indicative of cell wall/surface disruption, and with many fewer bacterial cells attached to the fungal elements (Figure 2b,d).

### 2.3. Inhibition of Mixed Biofilm on the Surface of the Functionalized Elastomer with AgNPs

**Figure 3:**
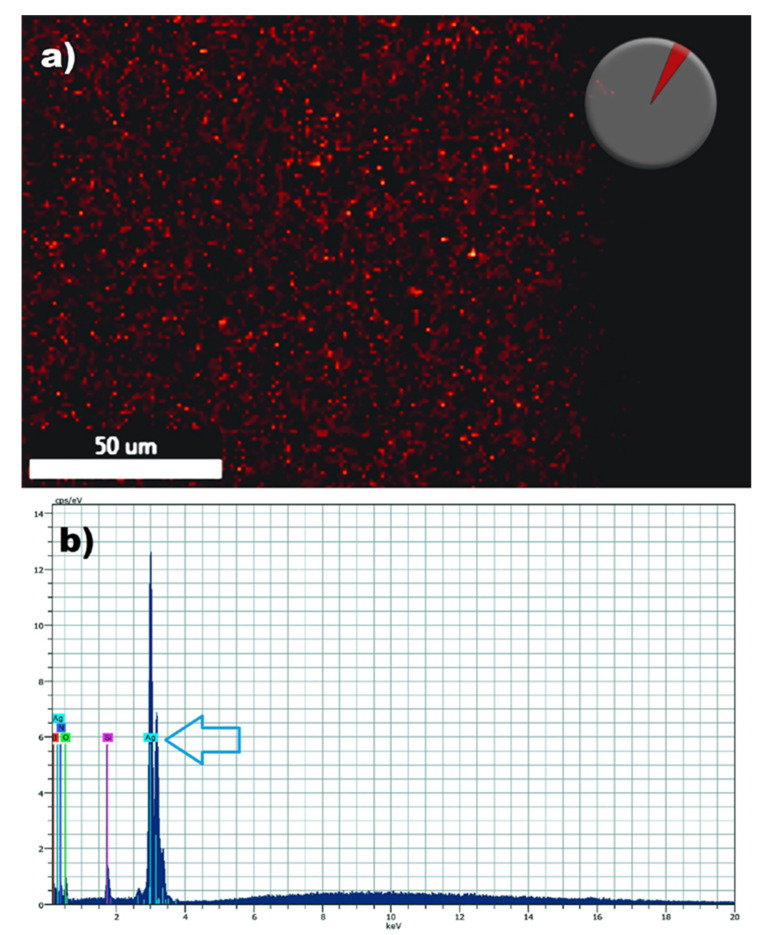
Detection of silver nanoparticles on the surface of the functionalized silicone elastomers sheets by Energy-dispersive X-ray spectroscopy (EDS). SEM/energy dispersive X-ray spectrometry demonstrates the presence of elemental silver signal on the surface of the functionalized elastomers. Red spots pinpoint Ag+ detection by spectral (**a**) and mapping acquisition in the silicone elastomer (**b**). The blue arrow points to the detection of a strong silver (Ag) signal.

Mixed biofilms formed by these two opportunistic pathogens often cause catheter-related blood stream infections associated with high mortality. Thus, after having established the potent activity of AgNPs against dual-species biofilms of *C. albicans* and *S. aureus* we posited that the functionalization of catheter materials with nonantibiotics such as our AgNPs could provide for an effective strategy to prevent mixed biofilm formation. In the first set of experiments we followed a procedure for the functionalization of medical grade silicone elastomers with AgNPs. The functionalization of the silicone elastomers with AgNPs (0.53 ppm) or without AgNPs (control) after thorough washing was demonstrated by the presence of AgNPs on the surface of the elastomers by using highly sensitive Energy Dispersive X-ray (EDX) microanalysis. Functionalized Silicone elastomers were scanned by spectral mapping and the red dots show the signal of AgNPs [25] demonstrating the presence of the signal of the element (Figure 3).

**Figure 4:**
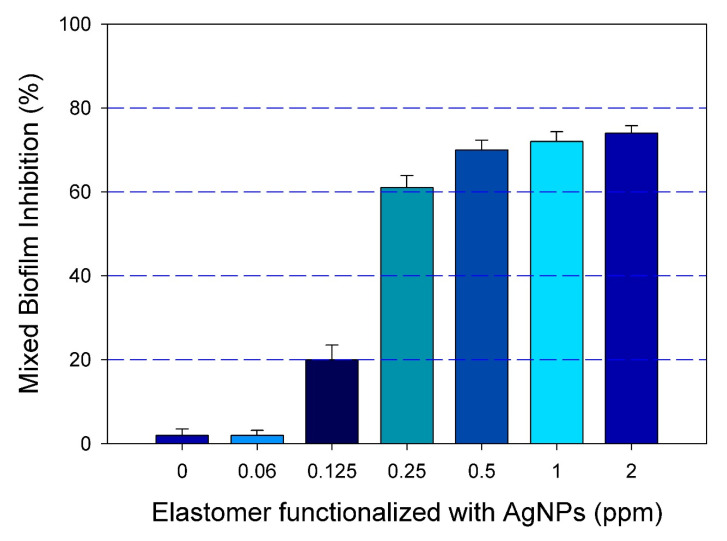
Inhibition of mixed biofilm growth on functionalized silicone elastomer sheets. The dose response inhibition was demonstrated by a resazurin reduction assay (% viability) on functionalized elastomers at different concentrations of AgNPs (0 to 2 ppm), showing the inhibition of the mixed biofilms in a dose response manner. Error bars represent standard deviation (SD) of the means.

Once the effective functionalization was demonstrated, we were interested in further investigating if functionalizing the surface of catheters with our positively charged AgNPs may lead to the inhibition of the mixed biofilm formation. To this extent, we used a modified assay in which functionalized silicone elastomers with different concentrations of AgNPs were used as the substrates for biofilm formation. Results indicated that, as compared to the untreated control, the functionalization of the elastomer with a range of concentrations of AgNPs (from 0.06 to 2.0 ppm) effectively inhibited the formation of mixed *C. albicans*/MRSA biofilm. Figure 4 shows the extent of mixed biofilm inhibition growth on silicone elastomers functionalized with different concentrations of AgNPs.

**Figure 5:**
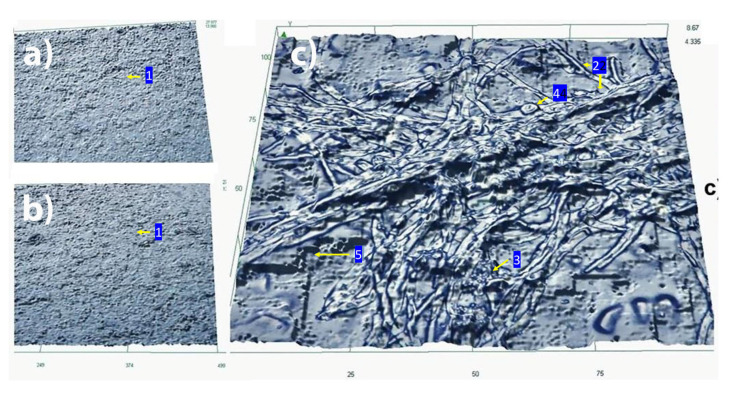
Functionalized silicone elastomers with AgNPs inhibit the formation of dual-species *C. albicans*/MRSA biofilms: visualization using opto-digital microscopy. 2D Images of Inhibition of a mixed biofilm by functionalized elastomers with AgNPs (0.53 ppm) (**a**) Functionalized elastomer inhibits the growth of the mixed biofilm. (**b**) Non-treated (functionalized) elastomer without biofilm growth as negative control showing the rugged surface of the elastomer (1). (**c**) Mixed biofilm growth on non-functionalized elastomer showing true hyphae (2), MRSA (3), yeast cells (4), and EPS (5).

High-resolution opto-digital microscopy was used to further document the inhibitory effect of functionalized silicone elastomer on mixed biofilm formation. After analyzing the effective dose response inhibition of the growth of the functionalized silicone elastomers by a viability assay (Figure 4) and to document high-resolution images in 2D of the growth inhibition of the mixed biofilm, we observed the functionalized (AgNPs 0.53 ppm) and non-functionalized silicone elastomers with an opto-digital microscope (Figure 5). As shown in Figure 5a, the functionalization of the same silicone elastomers with AgNPs (0.53 ppm) effectively prevented mixed biofilm formation. In contrast, mixed *C. albicans*/MRSA biofilm were able to grow on the surface of the control un-functionalized silicone elastomers, with abundant presence of fungal hyphal elements and MRSA colonies embedded within the extracellular matrix (Figure 5c).

## 3. Discussion

Biofilms are consortia of microbial cells attached to a substrate and embedded within a matrix of self-produced exopolymeric materials [12]. Both bacteria and fungi are capable of forming biofilms [26], and a majority of infections are associated with a biofilm aetiology. By virtue of their characteristics, the cells within these biofilms are protected against host immune mechanisms and also display high levels of resistance against most antibiotics [27,28]. Mixed fungal/bacterial biofilm infections are particularly hard to treat [10,29]. Together, *C. albicans* and *S. aureus* are responsible for a majority of opportunistic nosocomial infections [9,15], and they are often co-isolated from a host [13]. Frequently, these polymicrobial infections are associated with the formation of mixed biofilms in catheters and other indwelling devices, where *C. albicans* and *S. aureus* display a symbiotic relationship [16,30]. For example, MRSA resistance is enhanced within the mixed biofilm due to the protection by the fungal extracellular matrix, more specifically the secreted β-1,3-glucan component [9], and the invasive behavior of MRSA is facilitated by *C. albicans* leading to invasive infection in co-colonized patients. The ultimate effect is increased mortality and morbidity rates, with significant costs to our health care system [31].

Because cells within polymicrobial biofilms exhibit high levels of resistance to antibiotic treatment, alternative approaches are urgently needed to combat the threat of these biofilm-associated infections. Nanobiotechnology may provide a solution to this problem [32]. Our group has previously reported on the activity of pure positively charged silver nanoparticles synthesized by a novel microwave technique against single-species biofilms of *C. albicans* and MRSA [20,25]. Thus, we were interested in evaluating the activity of these AgNPs against dual-species *C. albicans*/*S. aureus* biofilms. Indeed, this was the case, and our results demonstrated a potent dose-response activity of AgNPs against preformed mixed *C. albicans*/MRSA biofilms. Interestingly, to our knowledge, there is only one additional recent report in the published literature on the use of nanotechnological approaches, more specifically curcumin loaded chitosan nanoparticles, against polymicrobial biofilms formed by these two microorganisms [33].

Catheter-related bloodstream infections are the cause of approximately one-third of all healthcare-acquired infection deaths [3]. The use of indwelling medical devices in hospitalized patients offers favorable conditions for microbial biofilm growth [4,5], and most often these two opportunistic pathogens (*C. albicans* and *S. aureus*) interact with each other and form mixed biofilms within this setting [9,15]. Antimicrobial-coated catheters have been proposed to decrease the chances of acquiring a catheter-related bloodstream infection, and an attractive alternative is to prevent colonization and biofilm formation by coating biomaterials with biofilm inhibitors [34,35]. The ideal antimicrobial catheter should offer a low-cost application technology and long-term broad-spectrum antimicrobial surface effect, without side effects or toxicity [36]. Nanotechnology-based approaches are designed to control and eradicate catheter-related bloodstream infections [24,37,38]. Therefore, after having established the potent activity of our AgNPs against these mixed fungal/bacterial biofilms in the standard 96-well microtiter plate model, we wanted to investigate their ability to inhibit mixed biofilm formation within a more clinically-relevant model, more specifically when used to functionalize the surface of silicone elastomers. The results from this set of experiments clearly indicated that functionalization of the elastomer with AgNPs resulted in significant inhibition of biofilm formation in a dose-response manner as compared to the untreated control. These results are consistent with those recently reported by our group on the inhibition of C. *auris* biofilm growth on the surface of a functionalized silicone elastomer with the same type of pure positively charged silver nanoparticles [24]. These results were further verified by using opto-digital microscopy. An opto-digital microscope incorporates conventional optical microscope, digital multimedia acquisition, and digital processing software to obtain highest quality images allowing to display the image details [39,40]. The resulting images corroborated the almost complete lack of biofilm formation in silicone sheets functionalized with AgNPs, as compared to control, non-functionalized, elastomers.

## 4. Conclusions

Overall, our results confirm the efficacy of AgNPs against mixed biofilms of *C. albicans* and *S. aureus*, and add to a growing body of evidence pointing to the activity of different types of nanoparticles against a variety of pathogenic microorganisms, including those capable of forming biofilms which are typically recalcitrant to clinically used antibiotics and for which there is an urgent need to develop preventive and therapeutic alternatives. This is where novel nanotechnological approaches, alone or in combination with conventional antibiotic therapy, may play an important role. Although our results indicate a strong potential of silver nanoparticles for the prevention and treatment of highly resistant polymicrobial biofilm-associated infections, we note that these are strictly in vitro results, and due to the complex interactions of silver with living tissues, important considerations regarding their biocompatibility and cytotoxicity need to be taken into account for their eventual pre-clinical and clinical development to combat the threat of mixed biofilm infections. 

## 5. Materials and Methods

### 5.1. Chemicals and Materials

All chemicals used in this study were purchased from Sigma-Aldrich (St. Louis, MO, USA), unless otherwise stated. Medical grade silicone elastomer sheets were purchased from Bentec Medical (Bentec Medical Inc, Woodland, CA, USA).

### 5.2. Microbial Strains, Media and Culture Conditions

To culture the mixed preformed biofilms in this study, the fungus *C. albicans* (SC5314), and the methicillin-resistant strain of *S. aureus* (MRSA TCH1516) were used. For long-term cryopreservation of *C. albicans* stocks were stored in 15% glycerol into an ultrafreezer (−80 °C), to maintain the yeast strain, yeast peptone dextrose (1% yeast extract, 2% peptone, 2% dextrose, YPD) agar plates were used and kept at 4 °C. Single yeast colonies were transferred from YPD plates into 10 mL of YPD liquid media for culturing *C. albicans*, which was routinely grown in an orbital shaker (180 rpm) at 30 °C overnight. Cells were pelleted by centrifugation at 5000× *g* for 5 min and the supernatant was decanted, then the resuspended cell pellet was washed twice in sterile phosphate-buffered saline (PBS, consisting of 137 mM NaCl, 2.7 mM KCl, 10 mM Na_2_HPO_4_ and 2 mM KH_2_PO_4_; pH 7.2) followed by a vortexing step of 2 min and centrifugation, dilutions (100-fold) of the suspended cells were prepared for biofilm growth and counted using a hemocytometer on a bright field microscope. Yeast cells were resuspended at a final concentration of 1.0 × 10^6^ cells/mL in the corresponding medium, to be seeded for biofilm formation in the 96-well microtiter plates (see below). The stock cultures of *S. aureus* MRSA TCH1516 were cryopreserved in aliquots at −80 °C in Brain Heart Infusion (BHI) broth (Difco, Becton Dickinson, Sparks, MD, USA) with 50% glycerol for long-term storage. A sterile applicator stick was used to streak out a small amount of inoculum from the frozen stock onto a selective chromogenic plate (BBL CHROMagar, BD Diagnostics, HD, Germany) and stored at 4 °C. Prior to each experiment, plates were incubated for 16–24 h at 37 °C, then a loopful of each stock culture was inoculated into 10 mL of Tryptic Soy Broth (TSB) liquid media at 37 °C for 24 h. The bacterial culture was sedimented by centrifugation (3600× *g* for 10 min at 4 °C), washed and resuspended in PBS and used for counting. The bacterial count of the inoculum was determined and resuspended to the final concentration (1 × 10^7^ CFU/mL) on BHI broth supplemented with 10% human serum on 96 well plates and incubated at 37 °C for 18 h. TSB and BHI have been previously determined to be optimal media for supporting both *C. albicans*/*Staphylococcus aureus* (dual species) biofilm [29].

### 5.3. Preparation and Characterization of AgNPs

AgNPs were obtained by a physical method (microwave irradiation-assisted synthesis) using the Ethos EZ microwave, a high-performance microwave digestion system (Milestone Inc., Shelton, CT, USA) as previously described by our group, resulting in the production of pure, round silver nanoparticles [20,24,25]. This MW technique allows for a fast rise in initial temperatures in the heat reaction. Briefly, 1.7 g of AgNO_3_ was dissolved in 10 mL of distilled water (DI) and treated by MW irradiation. The AgNO_3_ solution was continuously irradiated for 15 s at 1000 W. After MW irradiation, samples were cooled to room temperature (RT). The Transmission Electron Microscope (TEM) analysis with high-resolution images (JEM-2010, Jeol Ltd., Tokyo, Japan) was used to measure the average particle size distribution and shape of the AgNPs. AgNPs were in average 1–3 nm and rounded in shape (not shown). The physicochemical characterization of our pure AgNPs was performed as previously reported [20,24,25]. Briefly, the concentration of the solution after MW-irradiation was measured in part per million (ppm) by an atomic absorption spectrophotometer (AA-6200, Shimadzu Corporation, Kyoto, Japan); this technique is precise and sensitive therefore is the most used method in analytical measures for the metal concentration in a solution. To demonstrate the surface charge of the nanoparticles we used the Zetasizer Nano ZS (Malvern Instruments Ltd., Malvern, Worcestershire, UK) in solution at 25 °C. Over a time period of 120 h the Zeta potential (ZP) shifts to a positive surface charge indicating that this AgNPs become positively charged; as described in detail in our previously publication [20]. 

### 5.4. Formation of Mixed Fungal/Bacterial Biofilms in 96-Well Microtiter Plates

One hundred μL of the prepared dilutions with mixed microorganisms (1 × 10^6^ cells/mL for *C. albicans*, 1 × 10^7^ cells/mL for MRSA) in 1:1 *v*/*v* YPD/BHI added with 10% human serum were pipetted into each well of a sterile 96-well polystyrene tissue culture plates (Corning^®^ Incorporated, Corning, NY, USA). The plates were then incubated for 24 h at 37 °C. After incubation, the supernatant from each well was decanted and planktonic cells were removed by washing with 100 μL PBS. The viability of the cells within the biofilms was estimated by adding 100 μL of 1:10 *v*/*v* Presto Blue Cell Viability Reagent (Invitrogen, Carlsbad, CA, USA) in 1:1 *v*/*v* YPD/BHI media and incubated for 30 min at 37 °C. Finally, 80 μL from each well were transferred into a new 96-well plate for fluorescent readings. The microtiter plate reader (BioTek^®^ Synergy HT, Winooski, VT, USA) was set to measure fluorescence at 530/25 nm excitation and 590/35 emission.

### 5.5. In Vitro Activity of AgNPs against Mixed Fungal/Bacterial Biofilms 

AgNPs susceptibility testing was performed by adding AgNPs at two-fold serial dilutions concentrations to preformed mixed species biofilms grown in 1:1 *v*/*v* YPD/ BHI + 10% human serum, which were then incubated for an additional 24 h in the presence of AgNPs. Briefly, the wells of microtiter plates were seeded with mixed microorganisms as described above and incubated for 24 h to allow for biofilm formation. AgNPs were diluted in RPMI and added to the preformed biofilms (after tree PBS washings) at the following final concentrations: 2.0 to 0.015 ppm in YPD/BHI plus serum media, or without the AgNPs as the non-treated control and the medium alone as the blank control. After incubation for an additional 24 h, microtiter plates were washed and processed using the Presto Blue assay as described above. Additionally, IC_50_ values for AgNPs were determined by SigmaPlot^®^ plot analysis, using the four-parameter logistic nonlinear regression equation. All assays were performed in duplicate in independent experiments and were repeated at least three times.

### 5.6. Pretreatment, Functionalization and Characterization of Medical Grade Silicone Elastomers

For the pretreatment of the silicone elastomer, we used a previously reported protocol [41,42,43]. Briefly, medical grade silicone elastomer sheets were cut (1 cm^2^), washed with medical grade detergent, then all the detergent was washed off with several changes of distilled water, and the sheets were disinfected by steam sterilization (autoclave). The rubber sheets were treated overnight at 37 °C with sterile fetal bovine serum (FBS). Then, the elastomers were washed twice to rinse off the FBS and were placed into a sterile 48-well culture plate. The functionalization of the silicone elastomers with AgNPs was done as in [24]. Briefly, we added RPMI medium (2 mL) with either AgNPs at different concentrations (0.02 to 2 ppm) or without AgNPs (control) in a sterile 48-well microtiter plates, then incubated overnight at 37 °C. The pieces were washed three times with sterile phosphate buffered saline buffer to remove unattached nanoparticles. To confirm the presence of AgNPs attached on the silicone elastomers we used the spectral mapping acquisition by scanning electron microscopy/energy dispersive X-ray spectrometry (SEM/EDS) (Hitachi S-5500 SEM) as previously reported by our group [24]. This technique of elemental analysis is based on the generation of characteristic X-rays, is energy-specific to the silver atoms of the specimen by the incident beam of electrons. EDX microanalysis is used to qualitatively map whether elements in the spectrum are present at specific sites to be reported [25].

### 5.7. Inhibition of the Mixed Biofilms on the Surface of the Functionalized Elastomer by AgNPs

Silicone elastomers functionalized with AgNPs (2 to 0.02 ppm) and non-functionalized elastomers were tested to ensure the inhibition of mixed biofilm growth as in [24]. Briefly, RPMI media (1 mL) with 5 × 10^6^ cells/mL of *C. albicans* cocultured with 1 mL of 5 × 10^7^ MRSA in MOPS-buffered RPMI 1640 (pH 7.0), were placed in sterile 24-well culture plates, and incubated in an orbital shaker (100 rpm) at 37 °C. After 2 h (adhesion step), the rubber sheets were washed twice with 2 mL of PBS at room temperature to remove detached (planktonic) cells. Culture plates containing the elastomers were placed in an orbital rotatory shaker at 37 °C and 100 rpm overnight. The rubber sheets were washed thrice (PBS). The viability of cells was measured by Presto Blue Cell Viability Reagent as mentioned above, to calculate the biofilm inhibition in functionalized sheets as compared to the nonfunctionalized elastomer (control). All silicone elastomers were observed under opto-digital microscopy to corroborate the results (see below). All assays were performed in duplicate in independent experiments and were repeated at least three times.

### 5.8. SEM Assessments

Mixed biofilms were cultured at 37 °C for 24 h, as described above for observation in high resolution SEM as previously reported [20,44]. Briefly, on 48-well polystyrene tissue culture plates, mixed preformed biofilms were then treated with or without AgNPs (0.53 ppm) for another 24 h at 37 °C. After treatment, we gently washed the attached biofilm three times in sterile saline (PBS) and fixed with 4% formaldehyde (FA) and 1% glutaraldehyde (GA) at room temperature (RT) for 1 h. The fixed samples were gently washed three times in PBS and then post-fixed for 1 h at RT in 1% osmium tetroxide (OsO_4_) in a fume hood and then dehydrated through a series of ethanol concentrations (25%, 50%, 70%, 95% (10 min each), and absolute alcohol (for 20 min). The stained dehydrated mixed biofilm was then mounted on a 300-mesh carbon-coated copper grids to be observed by SEM in a Hitachi S-5500 (Hitachi Ltd., Tokyo, Japan). 

### 5.9. Opto-Digital Microscopy of the Mixed Biofilm on Silicone Elastomers

A previously reported protocol for the visualization of biofilms formed on silicone elastomers using opto-digital visualization 2D [39] was used to document the biofilm-inhibitory effect of the catheter materials functionalized by AgNPs, for which we used an opto-digital microscope (DSX 500, Olympus Corporation, Shinjuku City, Tokyo, Japan). The silicone elastomer sheets were pretreated and functionalized as indicated above. To ensure uniform biofilm formation on the functionalized or non-functionalized silicone elastomer sheets, 1 mL of a 5 × 10^6^ yeast cells/mL suspension of *C. albicans* was cocultured with 1 mL of 5 × 10^7^ MRSA in MOPS-buffered RPMI 1640 (pH 7.0), added onto the elastomers and incubated in an orbital shaker (New Brunswick Scientific, Edison, NJ, USA) at 100 rpm. After 2 h of incubation at 37 °C, the nonadherent cells were removed by gentle washing two times with sterile PBS, then elastomers were placed on sterile wells in a 24-well plate. After incubation for 24 h at 37 °C, elastomers were washed twice with sterile PBS and fixed with 4% formaldehyde (FA) and 1% glutaraldehyde (GA). After 1 h fixation at room temperature (RT) elastomers were observed on the surface to document the morphology of the mixed biofilm attached to the surface of the sheets by DSX500 High-resolution opto- digital microscope (ODM) to image and to visualize the biofilm growth or inhibition on the functionalized elastomers. We used the capability of this ODM to capture 2D images of the surface of the elastomers as ODM is a reliable method for biomedical exploration purposes [40].
